# Primary Xenografts of Human Prostate Tissue as a Model to Study Angiogenesis Induced by Reactive Stroma

**DOI:** 10.1371/journal.pone.0029623

**Published:** 2012-01-31

**Authors:** Viviana P. Montecinos, Alejandro Godoy, Jennifer Hinklin, R. Robert Vethanayagam, Gary J. Smith

**Affiliations:** 1 Department of Urology, Roswell Park Cancer Institute, Buffalo, New York, United States of America; 2 Department of Medicine, Roswell Park Cancer Institute, Buffalo, New York, United States of America; 3 Department of Hematology-Oncology, Faculty of Medicine, Pontificia Universidad Católica de Chile, Santiago, Chile; University of Illinois at Chicago, United States of America

## Abstract

Characterization of the mechanism(s) of androgen-driven human angiogenesis could have significant implications for modeling new forms of anti-angiogenic therapies for CaP and for developing targeted adjuvant therapies to improve efficacy of androgen-deprivation therapy. However, models of angiogenesis by human endothelial cells localized within an intact human prostate tissue architecture are until now extremely limited. This report characterizes the burst of angiogenesis by endogenous human blood vessels in primary xenografts of fresh surgical specimens of benign prostate or prostate cancer (CaP) tissue that occurs between Days 6–14 after transplantation into SCID mice pre-implanted with testosterone pellets. The wave of human angiogenesis was preceded by androgen-mediated up-regulation of VEGF-A expression in the stromal compartment. The neo-vessel network anastomosed to the host mouse vascular system between Days 6–10 post-transplantation, the angiogenic response ceased by Day 15, and by Day 30 the vasculature had matured and stabilized, as indicated by a lack of leakage of serum components into the interstitial tissue space and by association of nascent endothelial cells with mural cells/pericytes. The angiogenic wave was concurrent with the appearance of a reactive stroma phenotype, as determined by staining for α-SMA, Vimentin, Tenascin, Calponin, Desmin and Masson's trichrome, but the reactive stroma phenotype appeared to be largely independent of androgen availability. Transplantation-induced angiogenesis by endogenous human endothelial cells present in primary xenografts of benign and malignant human prostate tissue was preceded by induction of androgen-driven expression of VEGF by the prostate stroma, and was concurrent with and the appearance of a reactive stroma phenotype. Androgen-modulated expression of VEGF-A appeared to be a causal regulator of angiogenesis, and possibly of stromal activation, in human prostate xenografts.

## Introduction

Angiogenesis, the formation of new capillaries from pre-existing blood vessels, provides oxygen and nutrients for organogenesis during fetal development and homeostasis of adult tissue, as well as for survival and proliferation of cancer cells, functions crucial for organism and tumor growth [1], [2]. Increased microvessel density (MVD) in tumor tissue has been correlated with increased tumor stage, tumor grade, metastasis, and decreased cancer-specific survival. Therefore, MVD in prostate cancer (CaP) has been investigated as a potential prognostic marker for identification of patients at high risk of progression and recurrence after radical prostatectomy [3], [4], [5], [6], [7]. The viability, integrity and proliferative potential of human prostate endothelial cells, as in other organs, were demonstrated to depend on circulating androgens and on VEGF expression [7], [8], [9], [10]. However, the constitutive production of VEGF in human prostate appeared modulated by androgen, suggesting that AR-mediated expression of VEGF may regulate the balance between vascular stability and angiogenesis in the prostate vascular network [8], [11], [12].

Therapeutic inhibition of neo-vessel formation during progression of CaP offers hope for reducing morbidity and mortality. However, the promising results of anti-angiogenic therapeutics generated in animal models, or xenografts of human tumor cell lines transplanted into animals, have not predicted effectiveness in human patients. Angiogenesis within the tumor microenvironment is a complex process regulated by pro- and anti-angiogenic factors produced by both tumor epithelial cells and the stromal compartment [13], [14]. Consequently, conspicuous limitations of xenograft models based on implantation of permanent cultures of human tumor cells into immune-compromised mouse hosts include that the neo-vasculature of the xenografts is of mouse host origin, and that the neo-vessels develop and mature in response to a hybrid signaling milieu that emanates from both the host stromal microenvironment and the human tumor cells. These compromises are exacerbated in cell-line based prostate cancer xenografts by their inability to model the unique biological characteristics of human prostate vasculature, that human prostate endothelial cells demonstrate the highest proliferative index, and possibly the highest level of constitutive remodeling, of any vascular bed in the human body [8], and that the prostate endothelial cells express AR [15]. Therefore, a major factor that has limited development of appropriate independent, or adjuvant, anti-angiogenesis therapies for prostate cancer, or for most solid tumors, is a lack of pre-clinical models for analysis of human tumor vascular dynamics responding to an intact human tumor microenvironment. This study describes the dynamics of human angiogenesis that occurs in primary xenografts of human prostate tissue, either benign or prostate cancer tissue, transplanted to immuno-compromised (SCID) mice pre-implanted with a source of systemic androgen to maintain human serum levels of testosterone. Moreover, the human prostate primary xenograft model provides a unique tool for evaluation of the individual contributions of the endothelial compartment, the epithelial/cancer epithelial compartment, and the stromal compartment, to androgen-mediated homeostasis, or angiogenesis, of human prostate microvascular endothelial cells.

## Materials and Methods

### Clinical Specimens

Anonymous/de-identified human prostate and kidney remnant surgical tissue specimens were collected from patients, through a written consent, and processed in accordance with NIH guidelines for the use of human subjects, with approval by the IRB of Roswell Park Cancer Institute (RPCI). Benign prostate tissue for transplantation as primary xenografts was harvested from prostates removed surgically during cystoprostatectomy surgery, or from uninvolved areas of prostates harvested by radical prostatectomy. Normal kidney tissue was harvested from uninvolved areas of kidneys removed during radical nephrectomy surgery. CaP and RCC tissues for transplantation as xenografts were harvested from cores taken through palpable tumors removed during radical prostatectomy or radical nephrectomy surgery, respectively, and verified in a frozen section by a surgical pathologist before release from pathology. Tissue specimens were released by pathology for transplantation within two hours of the interruption of the blood supply to the prostate during surgery [16]. Pathology verified tissue specimens were submerged immediately in ice-cold ViaSpan solution (Barr Laboratories, Inc., Pomona, NY), and transported on ice for transplantation. An initial tissue (IT) specimen of at least 8 mm^3^ was removed as a control from each surgical tissue sample before transplantation, and was fixed in 10% formalin, and paraffin embedded for histological evaluation.

### Primary Xenografts of Intact Human Tissue

Xenografts were established by transplantation of intact pieces of prostate and kidney tissue as described previously [17], [18]. All experimental protocols that involved laboratory animals were performed in accordance with National Institutes of Health guidelines and were approved by the Institutional Animal Care and Use Committee of RPCI (IACUC #: 1044M). In brief, 3–5 days before transplantation of tissue, 3 month-old male SCID BALB/c mice were castrated and implanted subcutaneously with sustained-release testosterone pellets (12.5 mg; Innovative Research of America, Sarasota, FL) to maintain serum testosterone levels of ∼4.0 ng/ml throughout the study. Serum testosterone levels in the host were measured using a testosterone enzyme-linked immunosorbent assay (ELISA, Immuno-Biological Laboratories Inc, Minneapolis, MN). Surgical tissue specimens were cut into wedge-shaped pieces 2–3 mm in length and 1–2 mm in width, the tissue wedges dipped in Matrigel, the tissue pieces inserted individually into the subcutaneous compartment through small incisions on the right and left flanks of mouse hosts (4–5 xenografts per flank, with a maximal total of 8–10 xenografts per host), and the incision sites closed with Nexband tissue glue (Veterinary Products Laboratories, Phoenix, AZ). At selected times after transplantation, host animals were euthanized and the xenografts harvested for analysis: Days 1, 2, 3, 4, 7, 10, 14 and 30 after tissue transplantation. For analysis of the effect of circulating androgens on angiogenesis, xenografts also were implanted into castrated mouse hosts not implanted with supplemental testosterone pellets. Harvested xenografts either were frozen in OCT, or were fixed in 10% formalin for a minimum of 24 hrs, paraffin-embedded and sectioned (5.0 µm) onto ProbeOn Plus slides (Fisher Scientific International, Suwanee, GA).

### Immunohistochemical (IHC) Analyses

Formalin-fixed, paraffin-embedded sections of initial surgical tissue specimens (IT), or of primary xenografts, from benign and malignant human prostate tissue, benign human kidney tissue and RCC tissue were hydrated by sequential steps through Citri-solv (Fisher, Suwanee, GA) and a graded series of alcohol washes. After antigen retrieval with Citra Buffer (BioGenex, San Ramon, CA), endogenous peroxidase activity was blocked before incubation with primary antibody. After blocking, tissue sections were incubated overnight with antibodies against human CD31 (huCD31: 1∶40, Dako, Carpinteria, CA), huCD34 (1∶100, Neomarkers, Fremont, CA), msCD31 (1∶100, PECAM-1; BD PharMingen, Bedford, MA), von Willebrand (1∶100, Biogenex, San Ramon, CA), AR (N-20) (1∶100, Santa Cruz Biotechnology, Santa Cruz, CA), Ki-67 (1∶1500, Novocastra, New Castle, UK), Pan cytokeratin (1∶200, Sigma-Aldrich, Saint Louis, MO), prostate specific antigen (PSA) (1∶100 Dako, Carpinteria, CA) VEGF-A (1∶100, Neomarkers, Fremont, CA), VEGFR2 (1∶20, Sigma, Saint Louis, MO), α-smooth muscle actin (1∶200, Dako, Carpinteria, CA), Calponin (1∶5000, Sigma-Aldrich, Saint Louis, MO), Tenascin (1∶5000, Sigma-Aldrich, Saint Louis, MO), Desmin (1∶500, Sigma-Aldrich, Saint Louis, MO), Vimentin (1∶200, Dako, Carpinteria, CA), GLUT1 (1∶500, Alpha diagnostic, San Antonio, TX), HIF-1α and HIF-2α (1∶100, Novus Biologicals, Littleton, CO). All antibodies were diluted in 10 mM Tris-HCl buffer (pH 7.8) that contained 8.4 mM sodium phosphate, 3.5 mM potassium phosphate, 120 mM NaCl, and 1.0% BSA (w/v). After incubation with primary antibody, tissue sections were washed 3 times in Tris-HCl buffer (pH 7.8) for 10 min each, and incubated with HRP-conjugated anti-rabbit IgG or anti-mouse IgG (1∶100, DakoCytomation) for 2 h at room temperature. Peroxidase activity was developed using 100 mM Tris-HCl buffer that contained 3,3-diaminobenzidine tetra-hydrochloride (1.0 µg/ml, Sigma-Aldrich) and H_2_O_2_ (1.0 µl/ml, VWR International, West Chester, PA). Hematoxylin (Harris) was used as a nuclear counter-stain in tissue sections. Stained slides were dehydrated by sequential steps through a graded series of alcohol washes and Citrisolv (Fisher, Suwanee, GA), and were mounted using cover slips. For immuno-fluorescence studies, after incubation with primary antibody the specimens were incubated for 2 hrs with AlexaFluor488- or AlexaFluor594-conjugated affinity-purified donkey anti-rat or anti-mouse IgG (1∶200, Molecular Probes, Eugene, OR) secondary antibody at room temperature. DAPI (4′,6′-diamidino-2-phenylindole dihydrochloride) was used to counterstain nuclei for immunofluorescence studies. IHC or immunofluorescence staining in the absence of primary antibody, or using pre-immune serum, provided negative controls.

### Lectin Injection and Xenograft Tissue Fixation by Vascular Perfusion

Vasculature of primary xenografts of prostate tissue was visualized through intra-vital binding of fluorescently labeled lectins administered systemically, as described previously [19]. Mice were anesthetized with ketamine (100 mg/kg i.p.) plus xylazine (10 mg/kg i.p.), FITC-labeled *L. esculentum* lectin (100 µl 1.0 mg/ml in 0.9% NaCl; Vector Laboratories, Burlingame, CA) injected into the tail vein, and the lectin allowed to circulate through the mouse vascular system for 5 minutes. At the conclusion of the incubation, the chest was opened and the complete vascular volume replaced by perfusion with fixative (1% paraformaldehyde in PBS, pH 7.4) for 2 minutes at a pressure of 120 mmHg administered through an 18-gauge cannula inserted into the aorta via an incision in the left ventricle. Blood and fixative exited through an opening cut into the right atrium. Fixed xenograft tissues were removed, frozen in OCT, and processed for IHC.

### Determination of hypoxic areas

Animals were administered pimonidazole hydrochloride (Hypoxyprobe™-1, HPI, Burlington, MA) via intra-peritoneal injection (60 mg/100 gm body weight). One hour after injection, prostate xenografts were harvested, formalin fixed, paraffin-embedded, sectioned, and hypoxic areas visualized using IHC with a mouse monoclonal antibody that detected pimonidazole adducts (MAb1 1∶100, HPI, Burlington, MA). For positive controls of tissue hypoxia, xenografts harvested from animals previously administered pimonidazole were place in an hypoxia chamber (Invivo_2_ 400, Ruskinn, Pencoed, UK) for 1 hr before fixation.

### RT-PCR Analysis of Gene Expression

Total RNA from initial tissue specimens (IT) of human prostate tissue, and from prostate xenografts harvested on different days after transplantation, was prepared using the RNAeasy mini-kit (QIAGEN, Inc., Valencia, CA). Reverse transcription (RT) of mRNA was performed using the SuperScript III First-Strand kit (Invitrogen). Approximately 1.0 µl of reverse transcribed cDNA product was used as template in the Platinum PCR Supermix (Invitrogen) reaction mix that contained specific primer sets (200 nM). PCR products were separated using electrophoresis in 2% agarose gels and bands visualized with SYBR green gel stain (Molecular Probes). Glyceraldehyde-3-phosphate dehydrogenase (GAPDH) was used as a loading control in analytical gels. Primer sequences for the PCRs were: VEGF-A, forward 5′-ATGACGAGGGCCTGGAGTGTG-3′ and reverse 5′- CCTATGTGCTGGCCTTGGTGAG-3′; bFGF, forward 5′-TCACCACGCTGCCCGCCTTGC-3′ and reverse 5′-CAGTTCGTTTCAGTGCCACAT-3′; IL-8, forward 5′-ATGACTTCCAAGCTGGCCGTGGCT-3′ and reverse 5′- TCTCAGCCCTCTTCAAAAACTTCTC-3′; IL-6, forward 5′-AGCTCAGCTATGAACTCCTTCTC-3′ and reverse 5′-GTCTCCTCATTGAATCCAGATTGG-3′; TGF-β, forward 5′-AAGGACCTCGGCTGGAAGTG-3′ and reverse 5′-CCCGGGTTATGCTGGTTGTA-3′; IGF-1, forward 5′-CCTCCTCGCATCTCTTCTACCTG-3′ and reverse 5′- CTGCTGGAGCCATACCCTGTG -3′ and GAPDH, forward 5′-GGAAGGTGAAGGTCGGAGTCA-3′ and reverse 5′- GTCATTGATGGCAACAATATCCACT -3′.

### Digital Image Analysis

Digital images of immuno-histochemically and immuno-fluorescently stained sections of IT specimens, and of primary xenografts of benign and malignant human prostate and kidney tissues, were collected using a Hamamatsu 3CCD color camera mounted on an Axioskop microscope (Carl Zeiss, Inc., Thornwood, NY). Digital image analysis was performed using ImageJ software (Research Services Branch, National Institute of Mental Health, Bethesda, MD). Five fields (0.4 mm^2^) from each xenograft were analyzed to determine the average number of vessels per field (microvessel density = MVD) and the average number of vessels per field that contained at least one proliferating endothelial cell (expressing Ki-67).

### Statistical Analysis

Descriptive statistics, Student's paired, unpaired t tests, and data correlations were performed using Microsoft Excel 2000 (Microsoft Corp., Redmond, WA) or Prism 3 (GraphPad Software, Inc., San Diego, CA) using a 95% confidence interval. Average MVD, PMVD and MVA were graphed ± SE.

## Results

### Primary Xenografts of Human Prostate Tissue Undergo a Burst of Human Angiogenesis After Transplantation

**Figure 1:**
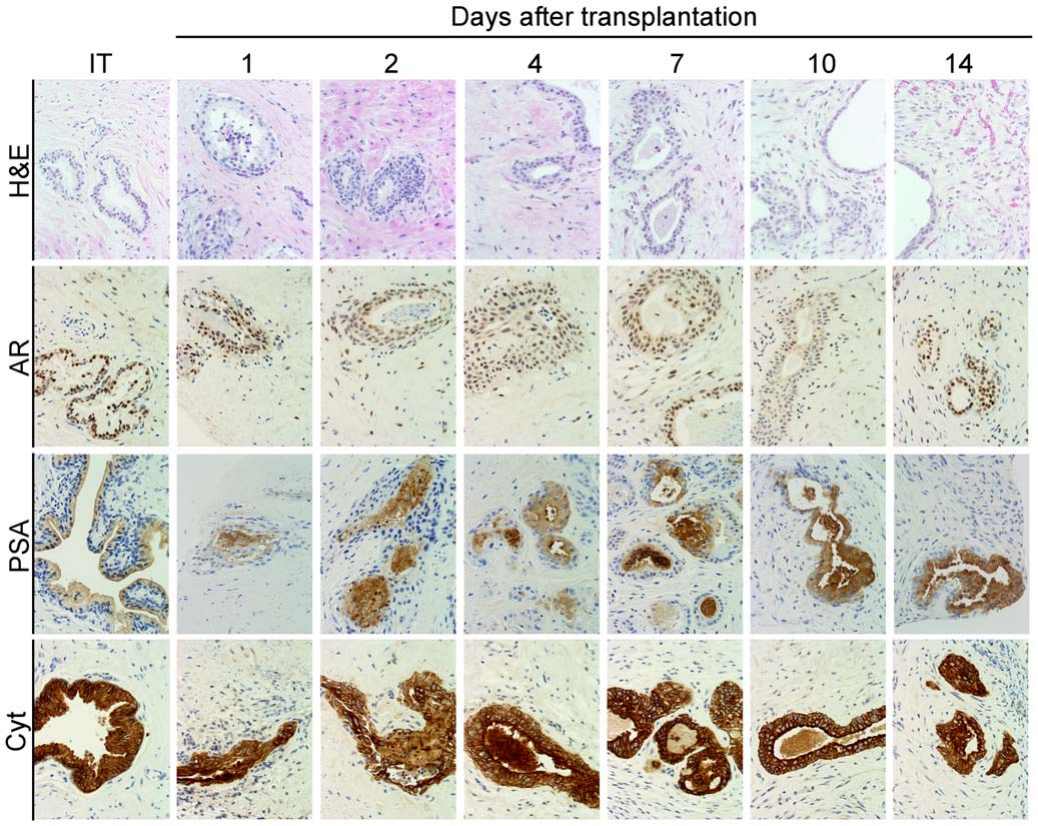
Primary xenografts of human prostate tissue maintain the *in vivo* tissue architecture and expression of key prostatic markers. Immuno-histochemical identification of protein expression of androgen receptor (AR), prostate-specific antigen (PSA) and pan-cytokeratin (Cyt) visualized by peroxidase staining demonstrated the level of expression remained constant over the fourteen days post-transplantation (1–14).

Mouse hosts were castrated and pre-implanted with slow release testosterone pellets, to establish human levels of serum testosterone, and subsequently were transplanted with human benign prostate tissue, or prostate cancer tissue. The morphology and function of the individual prostate cellular compartments were evaluated histologically and by IHC over the initial fourteen days after transplantation. The *in vivo* tissue architecture and inter- compartmental cell signaling milieu were preserved in primary xenografts of both benign and malignant prostate tissue for the entire duration of the study. The glandular epithelial cell compartment in the primary xenografts maintained expression of three markers of prostate epithelial cell differentiation, two of which depend on androgen-mediated signaling (AR and PSA) (Fig. 1). The lack of necrosis, and the maintenance of expression of AR and PSA, over the entire fourteen-day time-course after transplantation suggested the small size of the xenografts allowed efficient diffusion of oxygen, nutrients and androgens from the host mouse circulation into the xenografts, even before anastomosis of the xenograft and host vascular systems.

**Figure 2:**
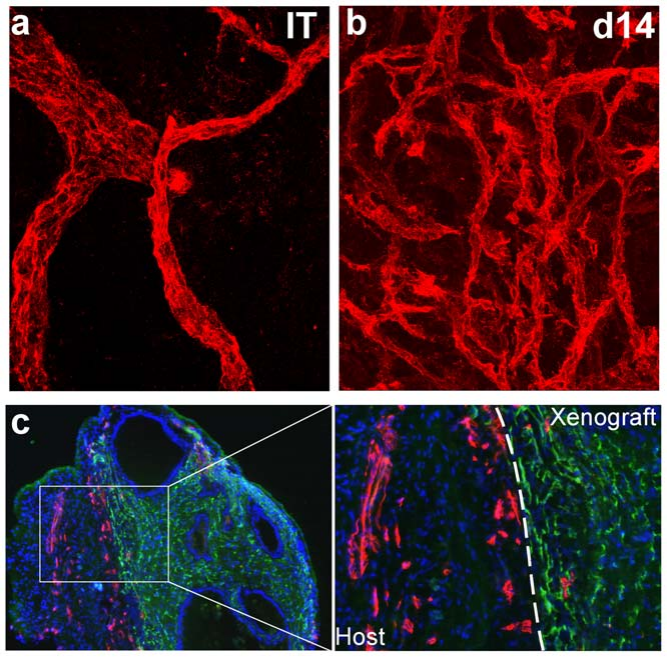
Primary xenografts of human prostate tissue undergo an explosive increase in human vessels over the initial 14 days after tissue transplantation. (**a–b**). Endothelial cells in primary xenografts of prostate tissue identified by human CD31 immuno-labeling and visualized by confocal laser scanning microscopy in initial tissue specimens (IT), and in primary xenografts of prostate tissue on Day 14 after tissue transplantation (d14). (**c**). Dual-immuno-histochemical staining with species-specific anti-human and anti-mouse CD31 antibodies in primary xenografts of prostate tissue on Day 14 after implantation. Human CD31 expression was visualized using FITC-labeled goat-anti-mouse IgG. Mouse CD31 expression was visualized using Cy3-labeled sheep-anti-rat IgG.

Three-dimensional reconstruction of serial optical sections collected by confocal laser scanning microscopic visualization of fluorescently-labeled anti-huCD31 antibody in primary xenografts of human benign and prostate cancer tissues demonstrated a dramatic increase in the number of vessels over the 14 days after transplantation compared to the corresponding initial tissue (IT) specimen before transplantation (Fig. 2a, b). The neo-vessels in prostate xenografts harvested on Day 14 after transplantation were abundant, tortuous, and of variable diameter (Fig. 2b). The small-to-medium caliber neo-vessels were dispersed throughout the xenografts, in contrast to vasculature in the IT specimen that predominantly was localized adjacent to glandular structures. Analysis of the species of origin of the endothelial cells in the neo-vessels involved in the angiogenic response during the 14 days after transplantation was performed by dual-immunostaining using species-specific antibodies for CD31 conjugated with different fluorochromes. The vast majority of vessels localized within prostate xenografts, whether endogenous, or newly formed during the angiogenic wave, were lined with human endothelial cells and lacked host endothelial cells (Fig. 2c, green staining). Vasculature of host origin, in contrast, was present at the periphery of the human prostate xenografts, localized predominantly to the cuff of compressed host tissue that surrounded the xenografts, with host vessels only occasionally penetrating into xenografts (Fig. 2c red staining).

### Time Course of Angiogenesis, Anastomosis and Maturation of the Newly Formed Vessels

**Figure 3:**
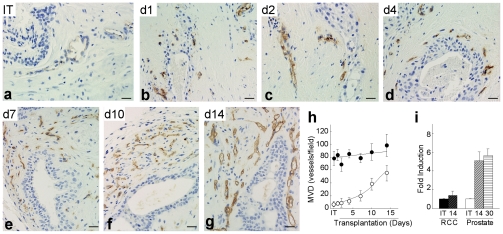
Time course of angiogenic activity in primary xenografts of human prostate. (**a–g**) Immuno-histochemical identification of blood vessels in initial tissue specimens before transplantation (IT), and in corresponding primary xenografts of prostate tissue during the fourteen days after transplantation (d1–d14). (**h**). Quantification of MVD in primary xenografts of prostate tissue, and RCC tissue, over the 14 days after tissue transplantation. MVD was quantitated by immuno-staining with anti-human CD31. (**i**). Quantification of MVD in prostate and renal tissue xenografts represented by fold-increase in human-CD31 positive cells in primary xenografts of prostate tissue, and RCC, on Day 14 (diagonal lines bars) and Day 30 (horizontal lines bars) after tissue transplantation, compared to the IT (solid bars). Bars = 50 µm.

Immuno-histochemical staining for the endothelial cell markers human CD31 (huCD31) (Fig. 3), CD34 and vWF (not shown) in primary xenografts of human benign prostate tissue and prostate cancer tissue demonstrated a dramatic increase in microvessel density (MVD) during the 14 days after transplantation (Fig. 3b–g) as compared to the MVD in the corresponding initial tissue specimen harvested before transplantation (Fig. 3a). In contrast to the peri-glandular localization of vessels in the initial tissue specimen, a time course analysis of neo-vascularization revealed that nascent vessels lined with huCD31 expressing cells were dispersed throughout the stromal compartment by Days 5–7 after transplantation, and the MVD increased progressively through Day 14. Quantification of MVD in xenografts demonstrated a consistent 5–7 fold increase in the MVD of human endothelial cell-lined vessels compared to the corresponding initial tissue specimen (Fig. 3h open circles, Fig. 3i, bars with diagonal lines). The marked increase in MVD occurred between Days 7 and 14 after transplantation; the MVD plateaued after Day 14 and remained at this level for at least 60 days (Fig. 3i, bars with horizontal lines). The angiogenic response in primary xenografts of human prostate tissue induced by transplantation, in both benign and malignant prostate tissue, was prostate tissue specific. Transplantation of fresh surgical tissues from benign kidney or RCC, a highly vascular tumor characterized by high rates of endothelial cell proliferation, was performed as a comparison for the angiogenic response observed in primary xenografts of the relatively avascular prostate tissue. However, neither xenografts of benign human renal tissue nor RCC tissue demonstrated an increase in MVD compared to the MVD of the initial tissue specimen (Fig. 3h,i - black circles and bars). Notably, the MVD in primary xenografts of benign renal tissue and RCC were similar on Day 14 after transplantation, but was approximately ten-fold higher than the MVD of the IT prostate tissue specimens. However, on Day 14 after transplantation, the MVD of prostate xenografts, as a result of vigorous angiogenesis, approached that observed for RCC xenografts (Fig. 3h).

**Figure 4:**
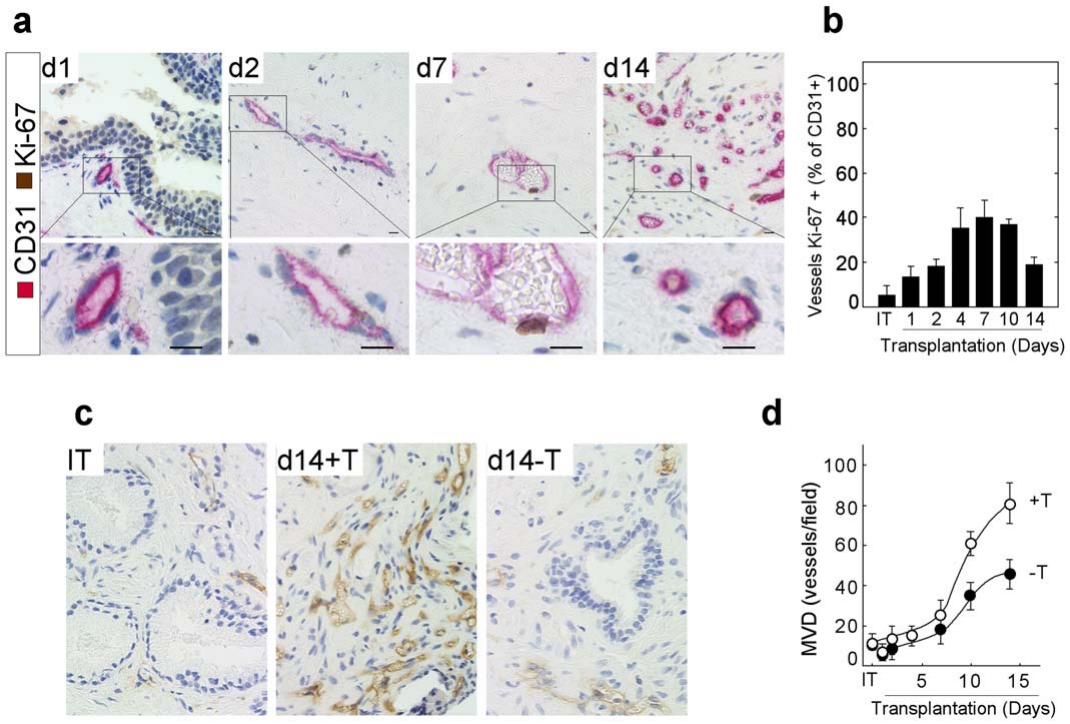
Dependence on androgen stimulation of proliferative activity of human endothelial cells in primary xenografts of human prostate tissue. (**a**). Co-localization of huCD31 (red) and Ki-67 (brown) protein demonstrated the increased presence of vessels with proliferatively active endothelial cells over the 14 days after tissue transplantation (d2–d14). (**b**). Quantification of the complete image set is presented in (**a**). Values were expressed as a percentage of total vessels that contained at least one Ki-67-positive endothelial cells. Bars = 10 µm. (**c**). Immuno-histochemical identification of human blood vessels in initial tissue (IT) specimens before transplantation, and in corresponding primary xenografts on Day 14 after tissue transplantation. The host mice were pre-implanted with, or not implanted with, sustained-release testosterone pellets. (**d**). Quantification of MVD in prostate xenografts over the 14 days after tissue transplantation into animals pre-implanted with (open circles), or not implanted with (closed circles), sustained-release testosterone pellets.

Proliferative activity of the human endothelial cells during the angiogenic wave was quantitated by IHC using co-localization of huCD31 and the marker of proliferation, Ki-67 (Fig. 4a). Endothelial cell proliferation was evaluated quantitatively during the 14 days after transplantation of xenografts into mouse hosts as the percent of vessels that contained at least a single Ki-67 positive endothelial cell (Fig. 4b). The peak of endothelial cell proliferation occurred between Days 4 and 10 after transplantation, with proliferation in the endothelial cell compartment returning to pre-transplantation levels by Day 14 (Fig. 4b). Therefore, the increase in MVD observed between Days 5 and 14 after transplantation was correlated temporally with an increased proliferative index in the human endothelial cells.

In the human prostate gland, angiogenesis has been correlated with the presence of androgen, which suggests AR-mediated regulation [12], [20], [21], [22]. The role of androgen stimulation in the angiogenic wave in primary xenografts of human prostate tissue was characterized by a temporal comparison of changes in MVD in xenografts transplanted to hosts pre-implanted with continuous release testosterone pellets, to changes in MVD in xenografts transplanted into castrate mouse hosts that were not pre-implanted with testosterone pellets. Figure 4c demonstrates a substantial difference in MVD at Day 14 post-transplantation between xenografts transplanted into mouse hosts with (d14+T), or without (d14-T), testosterone stimulation. Figure 4d presents a quantitative analysis of MVD of xenografts transplanted into host mice with (open circles), or without (closed circles), androgen stimulation. The MVD was decreased 60% on Day 14 in human prostate xenografts transplanted into hosts that lacked circulating testicular androgen.

**Figure 5:**
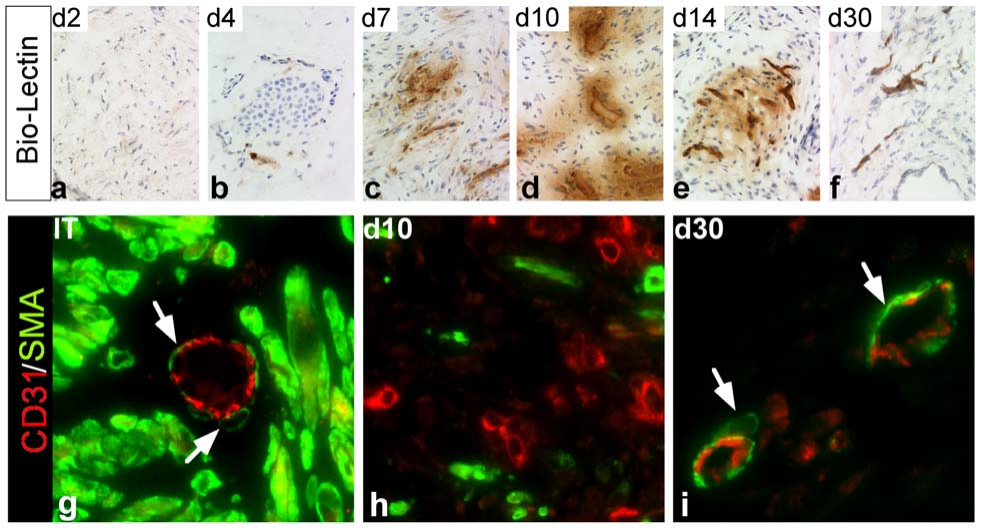
Vascular integrity and maturation of newly formed vessels in primary xenografts of human prostate tissue. (**a–f**). Immediately before xenograft harvest, the neo-vasculature in human prostate xenografts was labeled *in vivo* with biotin-conjugated lectin injected i.v. into the host mice. *In vivo* labeling studies demonstrated anastomosis of the prostate vasculature to the host vasculature by Day 7 after transplantation, and maturation of the human neo-vasculature by Day 30 after transplantation. (**g–i**). Confocal laser scanning microscopic visualization of dual-immuno-labeling of alpha-smooth muscle actin (αSMA, green) and huCD31 (red). Endothelial cells were associated with αSMA-positive peri-endothelial cells (indicated by arrowheads) on Day 30 post-transplantation.

The vascular network of primary xenografts rapidly become patent with the host vasculature, as demonstrated by labeling of endothelial cells of the xenograft vessels with biotinylated *Lycopersicon esculentum* lectin [23] administered systemically to the host by tail vein injection. On Day 4 post-transplantation, access of the angiogenic vessels to systemically available lectin was limited, as demonstrated by an absence of lectin within the xenografts (Fig. 5b). The number of lectin-stained vessels increased markedly between Days 7 and 10 after transplantation, which demonstrated the vascular network of the xenografts, including the vessels newly formed by angiogeneis, was patent with the host circulation and available to systemically injected lectin (Fig. 5c, d). Importantly, there was significant leakage of lectin into the interstitial tissue space surrounding the neo-vessels (Fig. 5c, d). Even as late as Day 14 after transplantation, when endothelial cell proliferation largely had ceased, the newly formed vessels leaked systemically administered lectins into the interstitial tissue space (Fig. 5e). However, on Day 30 after transplantation, lectin leakage into the interstitial space was absent, suggesting vascular maturation (Fig. 5f). Recruitment and association of alpha-smooth muscle actin (α-SMA) positive peri-endothelial cells (mural cells or pericytes) with the vascular endothelial cells is a marker of vascular maturation (lack of angiogenic activity) and vascular integrity [8], [11], [24]. Therefore, the absence of lectin leakage in the primary xenografts on Day 30 after transplantation was anticipated to be correlated with association of the neo-vasculature with α-SMA expressing cells. α-SMA expressing peri-endothelial cells were not associated with endothelial cells during the interval of active angiogenesis (Days 5 to 15 post-transplantation) (Fig. 5h) when the vessels had irregular, jagged contours, and leaked lectin into the interstitial spaces (Fig. 2b, 5c–e). In contrast, endothelial cells of the microvasculature in prostate xenografts on Day 30 post-transplantation were associated with α-SMA expressing mural cells (Fig. 5i), similar to the vasculature in the initial surgical tissue specimens (Fig. 5g). The decline in active endothelial cell proliferation, the association of the endothelial cells with mural cells, and the lack of lectin/fibrin/fibrinogen leakage into the interstitial tissue space suggested maturation and stabilization of the neo-vasculature occurred between Days 15 and 30 post-transplantation.

### The Angiogenic Burst in Primary Xenografts of Human Prostate Tissue is Preceded by Up-regulation of VEGF-A in the Stromal Compartment

**Figure 6:**
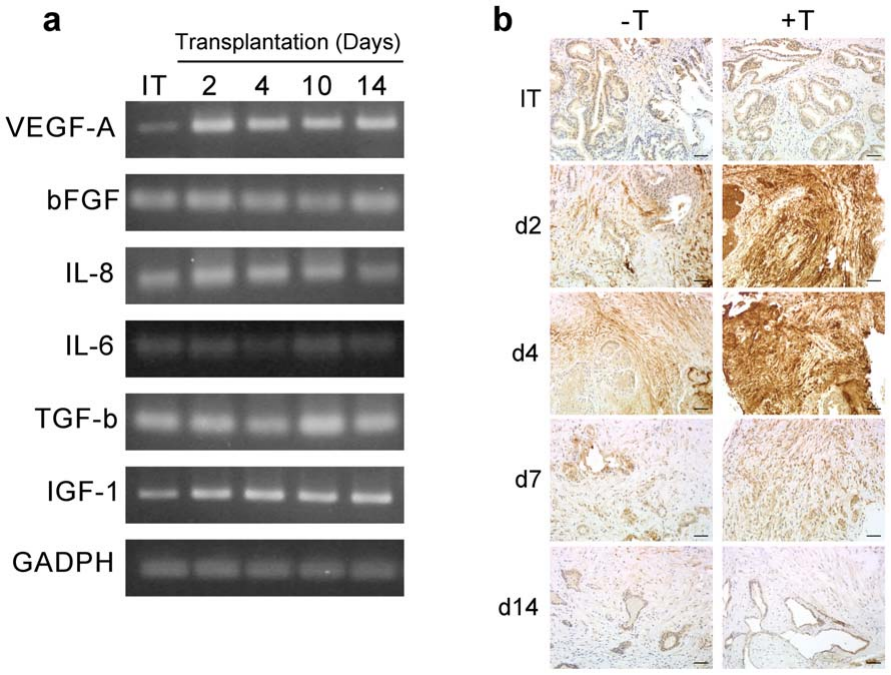
The angiogenic burst in primary xenografts of prostate tissue is preceded by androgen-modulated up-regulation of VEGF-A gene expression in the stromal compartment. (**a**). PCR analysis of expression of transcripts for pro-angiogenic factors in initial prostate tissue specimens before transplantation, and in corresponding primary xenografts after transplantation. Total RNA was extracted from initial prostate tissue (IT), and from prostate xenografts on different days after transplantation (d1–d14). GADPH was used as an internal control. (**b**). Immuno-histochemical identification of human VEGF protein in primary xenografts of prostate tissue over the 14 days after transplantation (d1–d14) in host mice pre-implanted with (+T), or not pre-implanted with (−T), sustained-release testosterone pellets. Bars = 50 µm.

The expression pattern of a select group of pro-angiogenic factors was characterized in human prostate xenografts using PCR analysis of mRNA isolated on different days after transplantation. Figure 6a presents the analysis of temporal changes after transplantation in mRNA expression of the pro-angiogenic factors VEGF-A, bFGF, IL-8, IL-6, TGF-β and IGF-1. Expression of all of the pro-angiogenic factors was detected in the matched initial tissue specimens before transplantation; however, transcripts were present at varying levels. Among these pro-angiogenic factors, VEGF-A demonstrated the strongest induction of expression in xenografts after transplantation into the androgenic environment of mouse hosts supplemented with exogenous testosterone. VEGF mRNA was expressed at very low levels in initial tissue specimens before transplantation, however, mRNA levels increased rapidly after transplantation, peaking on Day 2 (Fig. 6a).

IHC analyses of histologic specimens of initial prostate tissue specimens before transplantation, and the correlated prostate xenografts from the same surgical specimen harvested at different days post-transplantation, demonstrated the pattern of mRNA expression for the selected pro-angiogenic factors was reflected at the protein level. Consistent with the PCR analyses, minimal differences were found in protein levels for bFGF, IL-8, IL-6, TGF-β and IGF-1 in the human prostate xenografts compared to the initial tissue specimens (data not shown). However, levels of VEGF-A protein increased dramatically in response to transplantation. VEGF-A proteins levels peaked on Day 4 after transplantation, and returned to pre-transplantation levels by Day 8 (Fig. 6b). The rapid up-regulation of VEGF-A protein was localized largely to the stromal compartment of the human prostate xenografts; therefore the peak of stromal VEGF-A protein expression clearly preceded the wave of angiogenesis by human endothelial cells, which began after Day 5 (Fig. 3b; Fig. 3h, open circles). Consistent with previous reports that androgen regulated VEGF expression [12], [25], VEGF protein was induced to a substantially greater level in xenografts transplanted to mouse hosts implanted with testosterone pellets (Fig. 6b, +T).

**Figure 7:**
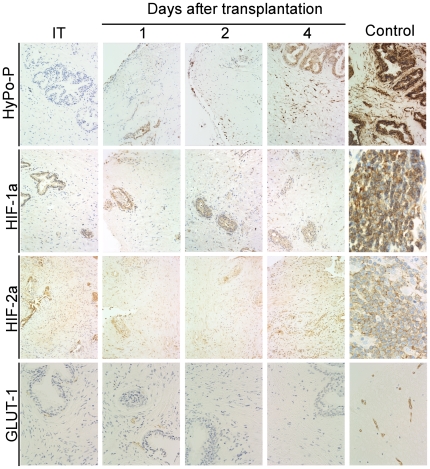
Determination of hypoxic areas, and of expression of HIF-1α, HIF-2α and GLUT1 in primary xenografts of human prostate. Animals were administered Hypoxyprobe-1 (HyPo-P, NPI Inc.) via *i.p* injection (60 mg/100 g body weight) on select days after tissue transplantation. One hour after injection, the prostate xenografts were harvested and hypoxic areas visualized using a monoclonal antibody specific for Hypoxyprobe-1. Immuno-histochemical identification of changes in human HIF-1α, HIF-2α and GLUT1 protein levels in primary xenografts of human prostate tissue over the 4 days after tissue transplantation (1–4). Hypoxic areas, and human HIF-1α, HIF-2α, and GLUT1 protein, were visualized using DAB and hydrogen peroxide.

Oxygen availability within tissue is an important regulator of angiogenesis; hypoxia is one of the most potent stimuli for VEGF expression and angiogenesis [26], [27]. The hypoxic response is mediated through stabilization of hypoxia inducible factors HIF-1α and HIF-2α, and results in up-regulation of expression of VEGF, the most prominent target gene of the HIFs [26], [27]. To determine whether the wave of VEGF expression, and subsequent angiogenic activity, in human prostate xenografts was associated with hypoxia that resulted from surgical excision and transplantation, mice were administered the hypoxia marker pimonidazole (Hydroxyprobe-1TM) by *i.p* injection at different times after transplantation of the prostate xenografts. After incubation to allow bio-distribution and tissue sequestration of pimonidazole in areas of hypoxia, xenografts were harvested and analyzed using IHC for localization of pimonidazole, and for analysis of protein levels of HIF-1α, HIF-2α and the hypoxia regulated gene, GLUT1. During the interval where stromal up-regulation of VEGF expression was apparent (Days 1 to 6 post-transplantation), areas of hypoxia were not observed in the xenografts, and HIF-1α/HIF-2α and GLUT1 protein levels remained constant (Figure 7). Consequently, hypoxia did not appear to drive the burst of VEGF expression in the primary xenografts.

### Increased Vascularization is Associated with the Emergence of a Reactive Stroma in Primary Xenografts of Human Prostate Tissue

In solid tumors, angiogenesis usually is accompanied by multiple other phenotypic changes in the tissue microenvironment. A “reactive stromal compartment” is a histological hallmark of invasive carcinomas, and may predict CaP recurrence-free survival [28]. Furthermore, the processes that lead to the formation of tumor-associated reactive stroma appear similar to those that operate at sites of wound healing [29]. To determine if the dramatic burst of human angiogenesis was associated with changes in stromal cell phenotype associated with transition to a reactive stroma, primary xenografts harvested on different days after transplantation were evaluated for the neo-expression of fibro-muscular markers and Masson's trichrome staining that differentially stains smooth muscle cells (red staining) and collagen fibers (green staining), indicators of the induction of a reactive stroma.

**Figure 8:**
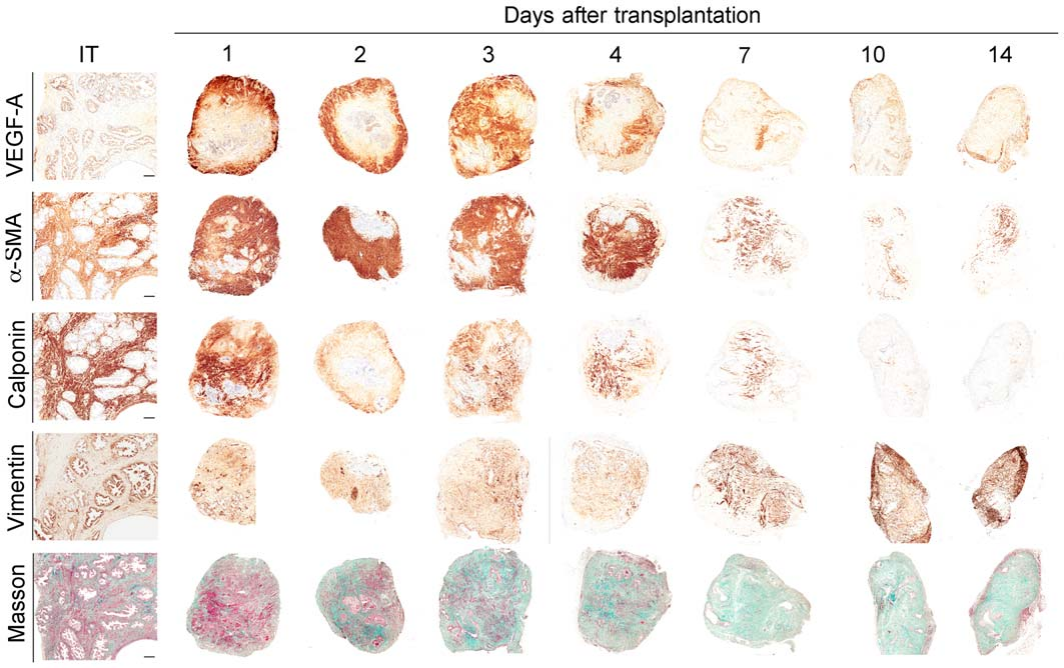
Induction of a reactive stroma in primary xenografts of human prostate tissue. Temporal changes of protein levels of VEGF, αSMA, Calponin and Vimentin were measured by IHC-staining, and of the presence of smooth muscle cells and collagen fibers was visualized by Masson's trichrome staining, over the 14 days following xenograft transplantation. α-SMA and Calponin are early and late markers of smooth muscle, respectively. Masson's trichrome identifies smooth muscle cells (purple) and collagen fibers (green).

Analysis of non-involved areas of fresh prostate surgical specimens (IT) showed broad expression of α-SMA and Calponin (early and late smooth muscle markers, respectively), and a mixture of red-staining smooth muscle cells and green-staining collagen fibers, which identify a fibro-muscular phenotype (Fig. 8, IT). Vimentin levels were low throughout the pre-transplantation stroma, with some vimentin positive cells observed adjacent to epithelial cell-lined acini. Starting on day 2 post-transplantation, concurrent with the up-regulation of VEGF-A expression in the stromal compartment, the smooth muscle cells observed in the stroma changed to a reactive stroma phenotype. By Day 14, the stroma showed a significant decrease, or complete loss, of markers of differentiated smooth muscle cells, and the extracellular matrix had become composed predominantly of collagen (green staining; Masson's trichrome). A limited analysis of the stromal compartment of prostate xenografts at Day 28 after transplantation demonstrated that, even though collagen deposition remained marked, expression of the fibroblastic markers characteristic of reactive stroma was lost, and the stroma had regained expression of the differentiated-stromal cell markers, a-SMA and calponin. At 28 days post-transplantation, expression of the markers of a fibro-muscular phenotype had recovered in the stroma of both benign and malignant prostate tissue xenografts to levels similar to those observed in the initial tissue (Fig. S1). Interestingly, these changes were prostate tissue-specific events since they were not observed in primary xenografts of human kidney tissue (Fig. S1). These results suggested that transplantation induced tissue-specific, and transitory, activation of the stroma of the primary xenografts of human prostate tissue, eliciting a transformation to a reactive stroma, with features similar to tumor-associated stroma.

## Discussion

The search for, and validation of, anti-angiogenic and chemotherapeutics treatments for localized prostate cancer has been handicapped by the lack of pre-clinical models that allow evaluation of candidate drugs in the context of an intact tissue microenvironment. The heterotypic signaling between cancer cells and the different cell types within the tumor microenvironment governs much of the biology of carcinomas, and experimental models that do not recapitulate this key component generate tumors that are biologically very different from those found in human cancer patients. For this reason, the ability of xenografts generated from cell lines propagated in culture to predict the response of tumors in patients to anti-cancer drugs is limited [30], [31]. This is particularly important in the arena of anti-angiogenic therapies. The essential role that VEGF-A plays in the neo-vascularization of malignant tissues has led to the emergence of anti-VEGF drugs as a panacea for the treatment of all tumors, regardless of the tissue of origin, based on studies in cell line-based xenografts. However, these promising results have not been observed in clinical studies [32], [33], [34].

Tumors in human patients exhibit intrinsic resistance to anti-VEGF therapy, or rapidly acquire resistance to anti-VEGF treatment, developing compensatory mechanisms to circumvent the anti-angiogenic agent. Mechanisms for adaptation to anti-VEGF treatment seem to be diverse, and new evidence suggests that tumor stromal cells (including endothelial cells) may be active players in the resistance to anti-VEGF therapy [35], [36], [37]. Consequently, the presence of mouse stromal and endothelial cell compartments in human cell line-based tumors represents a model not representative of human tumors *in situ*.

Recently, our group using the primary prostate xenograft model demonstrated that androgen deprivation, the standard treatment for locally advanced or metastatic prostate cancer, induced rapid involution and recovery of human prostate [38]. The acute apoptotic and reparative events in the human vasculature in primary xenografts induced by androgen deprivation were not observed in either the rat prostate or in human cell line-based xenografts, but is present in human subjects [39], [40]. The accurate recapitulation by the human prostate primary xenografts of the human prostate *in situ* could result in identification of more effective treatments for advanced CaP.

The human prostate primary xenografts utilized in these studies present many unique advantages as a pre-clinical model: 1) primary xenografts of benign and malignant human prostate are established with high efficiency from fresh human surgical tissue specimens; 2) a florid angiogenic response by endogenous human endothelial cells is elicited during establishment of xenografts of both CaP and benign human prostate tissue; 3) the xenografts maintain human prostate tissue architecture, and 4) after establishment, the proliferation/apoptotic rates in xenografts recapitulate those of the original tissue for at least 60 days after transplantation [18]. The angiogenic wave by human endothelial cells that produces the significant increase in MVD in primary xenografts human prostate [17] allows *in vivo* modeling of human angiogenesis/neo-vascularization occurring within an intact human prostate tissue microenvironment. However, a limitation of the primary xenograft model is that neither the benign nor cancer xenografts are amenable to serial passage from host to host animal. However, since the central focus of the model is to allow study of the homeostasis, and angiogenesis, of human prostate vasculature within an intact human prostate tissue microenvironment, the studies are not compromised by the limitation that they are confined to the primary (first) transplantation. Offsetting this limitation is the capacity to confirm the inter-patient universality of observations by analysis through xenografts derived from multiple independent patients.

The hypothesis that the increase in MVD was the result of angiogenesis by pre-existing human vessels is validated by the increased number of proliferating human endothelial cells observed during the initial days after transplantation, immediately preceding the increase in MVD. Recent reports have proposed that a significant portion of endothelial cells involved in formation of new vasculature are circulating endothelial stem/progenitor cells (EPCs) that are mobilized from the bone marrow (BM) by increased levels of circulating VEGF [41]. The circulating progenitor cells are recruited to growing or damaged vasculature, incorporate into the vasculature at the peripheral site, and differentiate into mature endothelial cells [42]. However, in the human prostate primary xenograft model, endothelial cells of host origin were not observed to be co-localized with the human endothelial cells in the neo-vasculature of the xenografts. This indicates, that in this model, BM-derived circulating EPCs of host origin do not contribute significantly to the genesis of the neo-vasculature in the xenografts. It is noteworthy that, despite the large increase in VEGF protein in the stromal compartment following transplantation, there was no concomitant increase in the level of circulating VEGF protein during the period of angiogenesis (data not show). This suggests a possible explanation for the lack of recruitment of host BM-derived endothelial precursors. However, human BM-derived endothelial precursors, or vascular progenitors resident in the human prostate tissue at the time of transplantation, may contribute to the observed neo-vascularization.

The lack of areas of hypoxia within prostate xenografts following transplantation, but before anastomosis of the xenograft microvasculature to the host vascular network, is a unique finding. The intra-vital lectin studies revealed that xenograft microvasculature was not patent with the host circulation until after Day 4 following transplantation. Nonetheless, the lack of necrosis in the center of the xenografts, and the maintenance of expression of the androgen-regulated proteins AR and PSA over the entire fourteen days time-course after transplantation, suggested that the small size of the xenografts (less than 2 mm cubed) allowed efficient diffusion of oxygen and androgen from the host circulation into the xenograft before anastomosis. Additionally, the architectural pattern of the up-regulation of VEGF-A expression (from the periphery of the xenografts toward the center) also suggested that the signals that induced the burst of VEGF expression were not hypoxia mediated.

An alternative mechanism for regulation of VEGF expression in the prostate xenografts was suggested by reports that VEGF expression in human prostate is androgen-regulated [8], [11], [12]. The prostate gland is an androgen-sensitive organ, and many of the cell types in prostate demonstrate AR-transactivation of gene transcription, including endothelial cells [15]. Consequently, perturbation of the androgenic milieu of the prostate may result in modulation of the entire tissue microenvironment, including endothelial cell homeostasis and angiogenesis [43]. A direct role for androgen in modulation of stromal VEGF expression was validated by our demonstration that VEGF expression by stromal cells was induced to a substantially greater level in xenografts transplanted to mouse hosts implanted with testosterone pellets (Figure 6B). Furthermore, we reported recently that human prostate endothelial cells express functional AR, and that androgen modulates proliferation of human prostate endothelial cells through an AR-dependent mechanism [15]. Consequently, androgens may affect proliferation of human prostate endothelial cells *in vivo* by both direct (endogenous AR-mediated) and indirect (paracrine VEGF from stroma) mechanisms. This hypothesis is reinforced by our observations that endothelial cells from human kidney tissue lack of expression of AR, and kidney xenografts do not demonstrate of up-regulation of VEGF after transplantation.

VEGF-A over-expression by tumor cells leads to the formation of nascent tumor blood vessels of exaggerated size, tortuosity, and permeability [8], [44], [45]. VEGF-A over-expression also is capable of inducing formation of tumor-like blood vessels in normal tissue in the absence of tumor cells [46], [47], [48]. The neo-vessels produced in the primary prostate xenografts during the angiogenic wave that follows the induction of stromal VEGF-A expression are abnormal, resembling those observed in malignant tissues [8], [44], [45], [46]. Furthermore, cessation of stromal VEGF expression preceded the cessation of vascular leakage and the recruitment of mural cells to the endothelial cells associated with maturation and stabilization of neo-vasculature, consistent with the pro-angiogenic properties of VEGF [49]. Consistent with these observations, reports suggest that tumor vessels that lack adequate pericyte coverage are more vulnerable to anti-angiogenic therapy [8], [50], and that dual targeting of endothelial cells and pericytes improves therapeutic efficacy in a variety of mouse tumor models [50], [51], [52]. Consequently, the well-defined time windows in the human prostate xenografts for angiogenesis, anastomosis to the host vasculature, and maturation of the human vasculature, marked by loss of leakage and association with mural cells, suggests the model may provide a unique pre-clinical tool to study further the relationship between vessel maturation and the anti-tumor efficacy of candidate anti-angiogenic agents, and combinations of agents.

A key role for the stromal compartment in mediation of angiogenesis in the human prostate is suggested by this study. Studies of human breast, colon, and prostate cancers have demonstrated that increased MVD within the stromal compartment is a common component of cancer progression [53], [54], [55], [56]. Additionally, the stromal compartment of these cancers exhibit a reactive stromal phenotype associated with expression of a novel spectrum of extracellular matrix (ECM) components compared to the stroma of benign tissue. The term “carcinoma associated fibroblasts” (CAFs) has been used to describe this reactive phenotype [53], [54]. However, little is known about the molecular events that govern the conversion of a benign fibro-muscular stroma into a carcinoma-associated stroma. Analysis of gene expression patterns of CAFs demonstrated strong similarities between these cells and the fibroblasts present at sites of active tissue repair, such as in the wound healing process [57], and experimental models support a role for TGF-β signaling in this progression [56], [58]. During the establishment of primary xenografts of both CaP tissue and its benign counterpart, increased collagen deposition and vimentin expression were observed, with a loss of α-SMA, Calponin and Desmin expressing cells, features associated with the appearance of a reactive stroma phenotype in human prostate tissue *in situ* [28]. The common response of the stromal compartment of both benign and malignant prostate tissue suggests that up-regulation of VEGF expression is an innate response to stress, possibly similar to the well-characterized stromal response to tissue damage. Increased expression of VEGF has been reported in other models of transplantation, such as in mouse allografts, and at sites of transplantation of tissue from human donors [59], [60], [61]. Additionally, breast cancer patients undergoing surgery for removal of primary tumors can exhibit a burst of VEGF synthesis at the wound site after surgery; importantly, over-expression of VEGF in the healing breast was localized mainly in the stromal compartment [62]. Furthermore, the potential role of VEGF as a promoter of tumor angiogenesis, as well as tumor stromagenesis, has been suggested previously [63]. Even though our data do not demonstrate that VEGF-A up-regulation is the sole causal factor driving the transition from a benign fibro-muscular phenotype to a reactive stroma, the link cannot be completely discarded based on the facts that: 1) VEGF expression was drastically, but not totally, reduced in the absence of androgen and that the threshold concentration of VEGF necessary to induce angiogenesis, or stromagenesis, could be different in the absence of androgen, and 2) the pattern of expression of the stromal markers reverted to the pattern observed in the initial prostate tissue concurrent with the cessation of angiogenic activity.

### Summary and Conclusions

**Figure 9:**
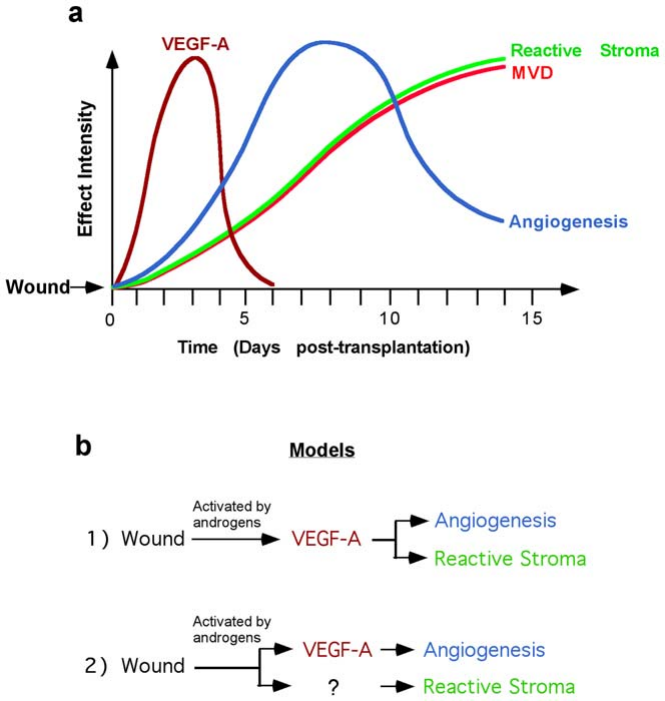
Schematic representation of timeframe of the transplantation-induced biological processes in primary xenografts of human benign and malignant prostate tissue. (**a**) Temporal changes of VEGF-A expression (brown line), angiogenesis (blue line), microvessel density (red line) and expression of a reactive stroma phenotype after xenograft transplantation. (**b**) The data from graph (a) suggests two hypothetical models of the cause-effect relationship of VEGF-A expression with angiogenesis and reactive stroma generation in primary xenografts of human prostate tissue.

During the course of this analysis a study was reported by Pinto *et al.* [64] that VEGF secreted by a reactive stromal cell line (BJ3Z) isolated from mammary gland enhanced angiogenesis and hormone-independent growth of estrogen receptor-positive breast cancer xenografts. In our study, however, transplantation of both human benign and malignant prostate tissue set in motion a chain of events within the stromal compartment that led to generation of a reactive stroma and angiogenesis (Fig. 9). The burst of stromal VEGF-A was unexpected, and suggested that up-regulation of VEGF-A expression was an innate response of the human prostate stroma cells, similar to the stromal response to tissue damage [29], a response that possibly could contribute to the appearance of the reactive stroma. Interestingly, the angiogenic response in primary xenografts of human prostate tissue was “activated” by, but not “dependent” on, the presence of circulating androgens. The proposed sequence and timeframe for these processes were: 1) up-regulation of VEGF (peaking at Days 2–4 after transplantation), 2) angiogenesis (peaking at Day 7 after transplantation), and 3) generation of reactive stroma (plateauing after Days 10–14 after transplantation) (Fig. 9a). Based on this reproducible sequence of events, two hypothetical models can be proposed to explain the role of VEGF-A in the temporal occurrence of these processes (Fig. 9b). In the first model, tissue transplantation induces up-regulation of VEGF-A directly, which activates both angiogenesis and the appearance of reactive stroma. In the second model, generation of reactive stroma by prostate tissue occurs by a tissue transplantation-induced mechanism that is completely independent of the up-regulation of VEGF-A and/or mechanisms stimulating angiogenesis. In the second model, the temporal difference between tissue transplantation and the appearance of a reactive stroma could suggest participation of a transplantation-induced unknown intermediary factor(s) in the generation of reactive stroma. Elucidation of the microenvironmental mechanism(s) responsible for initiating and driving the angiogenic response, and/or reactive stroma generation, might have broad application in understanding the development and progression of CaP, and other tumors. However, more analyses are necessary to elucidate the role of VEGF signaling in generation of a reactive stroma, studies that could identify new therapeutic targets to effectively counteract progression of CaP, and potentially other hormonally responsive tumors.

## Supporting Information

Figure S1 **Induction of a reactive stroma in primary xenografts of human prostate and kidney tissues.** Temporal changes in protein levels of αSMA and Calponin were measured by evaluation of IHC-staining and collagen fibers visualized by Masson's trichrome staining in initial prostate tissue (IT) and prostate xenografts on different days after transplantation (d4, d14, d28).
(TIF)
