# Supplementary figures and images for: Primary Xenografts of Human Prostate Tissue as a Model to Study Angiogenesis Induced by Reactive Stroma
Source: PLoS One. 2012 Jan 31;7(1):e29623. doi: 10.1371/journal.pone.0029623 (PMC3269421; doi:10.1371/journal.pone.0029623)

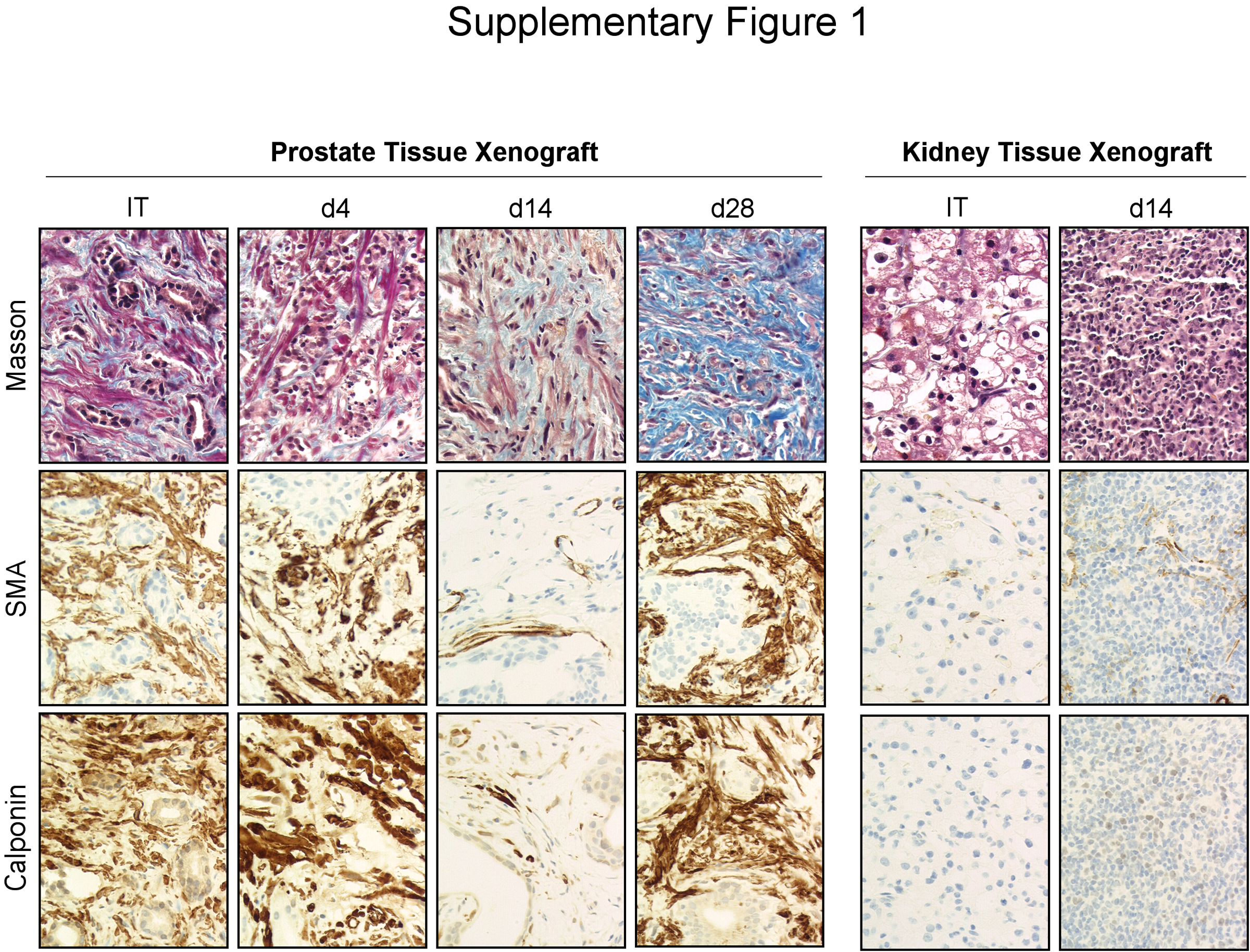

Supplement: Figure S1 — Induction of a reactive stroma in primary xenografts of human prostate and kidney tissues. Temporal changes in protein levels of αSMA and Calponin were measured by evaluation of IHC-staining and collagen fibers visualized by Masson's trichrome staining in initial prostate tissue (IT) and prostate xenografts on different days after transplantation (d4, d14, d28). (TIF) [file pone.0029623.s001.tif]
